# HuR Plays a Positive Role to Strengthen the Signaling Pathways of CD4^+^ T Cell Activation and Th17 Cell Differentiation

**DOI:** 10.1155/2021/9937243

**Published:** 2021-08-04

**Authors:** Shiguang Yu, Morgan Tripod, Ulus Atasoy, Jing Chen

**Affiliations:** ^1^Department of Neurology, Thomas Jefferson University, Philadelphia, PA 19107, USA; ^2^Arkansas Bioscience Institute and Department of Biological Science, Arkansas State University, Jonesboro, AR 72467, USA; ^3^Division of Allergy and Immunology, Department of Internal Medicine, University of Michigan, Ann Arbor, MI 48109, USA

## Abstract

After antigen and/or different cytokine stimulation, CD4^+^ T cells activated and differentiated into distinct T helper (Th) cells via differential T cell signaling pathways. Transcriptional regulation of the activation and differentiation of naïve CD4^+^ T cells into distinct lineage Th cells such as Th17 cells has been fully studied. However, the role of RNA-binding protein HuR in the signaling pathways of their activation and differentiation has not been well characterized. Here, we used HuR conditional knockout (HuR KO) CD4^+^ T cells to study mechanisms underlying HuR regulation of T cell activation and differentiation through distinct signaling pathways. Our work showed that, mechanistically, HuR positively promoted CD3g expression by binding its mRNA and enhanced the expression of downstream adaptor Zap70 and Malt1 in activated CD4^+^ T cells. Compared to WT Th0 cells, HuR KO Th0 cells with reduced Bcl-2 expression are much more susceptible to apoptosis than WT Th0 cells. We also found that HuR stabilized IL-6R*α* mRNA and promoted IL-6R*α* protein expression, thereby upregulating its downstream phosphorylation of Jak1 and Stat3 and increased level of phosphorylation of I*κ*B*α* to facilitate Th17 cell differentiation. However, knockout of HuR increased IL-22 production in Th17 cells, which was due to HuR deficiency in reducing IL-22 transcription repressor c-Maf expression. These results highlight the importance of HuR in TCR signaling and IL-6/IL-6R axis driving naïve CD4^+^ T cell activation and differentiation into Th17 cells.

## 1. Introduction

After antigen stimulation, CD3 complex associated with T cell receptor (TCR) and the z chain (zeta-chain) activates and enhances the signaling cascades that determine T cell fate [1]. CD4^+^ T cells activate and differentiate into various effector subsets by the production of distinct cytokines and by their distinct effector functions. Zeta-chain-associated protein kinase (Zap70) is one of the two members of the cytoplasmic Syk tyrosine kinase family and recruited to the TCR/CD3 complex and activated. Zap70 plays an essential role in T cell development and activation [1, 2], which is dependent on Lck to initiate the downstream signaling pathway [3]. Malt1 plays an important role in T cell activation and proliferation [4, 5]. TCR engagement leads to the formation of an oligomeric CBM complex (Carma1, Bcl10, and Malt1 proteins), which is required for activation of downstream canonical NF-*κ*B signaling [6]. In addition, Malt1 has paracaspase activity that cleaves specific protein substrates, such as RelB and A20, which then facilitate T cell activation [7, 8]. Previous studies provided evidence that Malt1 is essential for development of pathogenic T helper 17 (Th17) cells during experimental autoimmune encephalomyelitis (EAE) induction [9].

Although IL-17 (IL-17A) is the signature cytokine of Th17 cells, Th17 cells also produce IL-17F, GM-CSF, IL-22, and IL-21 [10]. IL-17 acts *in vitro* and *in vivo* as a potent inflammatory cytokine [11]. IL-17-deficient mice are resistant to the development of adjuvant-induced arthritis [12]. Similarly, IL-17-deficeint mice develop experimental autoimmune encephalomyelitis (EAE) with delayed onset and reduced severity [13].

Naïve CD4^+^ T cells differentiate into Th17 cells when activated with antigen in the presence of TGF-*β*, IL-6, and IL-1*β* [14]. IL-23 cannot drive the differentiation of naïve CD4^+^ T cells into Th17 cells, but it can promote Th17 cell proliferation and pathogenicity [10, 15, 16]. Like the transcription factor T-bet for Th1 cells and Gata3 for Th2 cells, STAT3 and ROR*γ*t are responsible for promoting Th17 cell differentiation [17, 18]. Further studies also showed that other multiple transcription factors are involved in Th17 cell differentiation, including Irf4, Runx1, c-Maf, and Ahr [19].

Recent studies progress in understanding of the transcriptional regulation of Th17 cell activation and differentiation; however, it remains largely unknown how CD4^+^ T cell activation and differentiation are posttranscriptionally regulated by RNA-binding proteins. Considering the importance that posttranscriptional regulation plays an important role in modulating gene expression for quick responses to environmental stimuli [20, 21], it is crucial to study posttranscriptional gene regulation in naïve CD4^+^ T cell activation and differentiation into Th17 cells. It is well known that HuR (EVLAV) posttranscriptionally regulates gene expression in cancer cells [22, 23], and HuR targets many mRNAs that contain the adenylate-uridylate-rich elements (ARE) in 3 untranslated regions (3′UTR) [22], which encode proteins with role in cell proliferation and survival [23]. In immune cells, HuR posttranscriptionally regulates cytokine expression such as IL-13, TNF-*α*, GM-CSF, and IL-17 and chemokine CCR6 expression [24–28]. Our current study revealed that HuR positively regulates CD4^+^ T cell activation by promoting CD3g and downstream Zap70 expression. Knockout of HuR impaired the level of phosphorylation of Stat3 in Th17 cells. Mechanistically, HuR stabilized IL-6R*α* mRNAs and prolonged their half-lives, which, in turn, enhanced the downstream phosphorylation of Jak1 and Stat3 to drive Th17 cell differentiation. Although recent work shows that HuR promotes Stat3 translation in muscle cells during inflammation-induced muscle wasting [29], knockout of HuR did not impair total Stat3 expression in Th17 cells. Interestingly, IL-22 levels increased in *in vitro-*cultured HuR KO Th17 cells, which was consistent with the decreased expression of c-Maf in HuR KO Th17 cells, as c-Maf has been reported as IL-22 transcription repressor [30]. Thus, HuR plays a positive role to strengthen the signaling pathway of CD4^+^ T cell activation and Th17 cell differentiation.

## 2. Materials and Methods

### 2.1. Animals

HuR^flox/flox^ mice were generated as previously described [31]. Eight- to ten-week-old control (HuR^flox/flox^) male and female mice (WT) and HuR conditional knockout mice (OX40-*cre+/-* HuR^flox/flox^) (KO) were used. All mice are on the C57BL/6 background and were bred at the animal facilities of Arkansas State University and Thomas Jefferson University. Animal experiments were approved by the Institutional Animal Care and Use Committee (IACUC) of Arkansas State University and Thomas Jefferson University and done according to guidelines of NIH. Both male and female mice were used in the experiments.

### 2.2. Isolation and Differentiation of CD4^+^ T Cells *In Vitro*

Naive CD4^+^ T cells were purified from splenocytes using CD4-negative selection kits (Stem Cell Inc., Canada) following the manufacturer's protocol. Cells were cultured as described before [24, 25]. For Th0 cell culture, WT and HuR KO CD4^+^ T cells were activated with plate-bound anti-CD3 (3 *μ*g/ml) and anti-CD28 (2 *μ*g/ml) for 5 days in 12-well plates at 3 × 10^6^ cells/ml. For Th17 polarization, WT and HuR KO CD4^+^ T cells were activated with plate-bound anti-CD3 (5 *μ*g/ml) and anti-CD28 (2 *μ*g/ml) for 5 days in 12-well plates at 3 × 10^6^ cells/ml. TGF-*β* (1-3 ng/ml), IL-6 (20 ng/ml), IL-23 (20 ng/ml), anti-IFN-*γ* (10 *μ*g/ml), and anti-IL-4 (10 *μ*g/ml) were used additionally in Th17 cell culture. For IL-2 neutralization experiment, anti-IL-2 (30 *μ*g/ml) was added in Th17 polarization cytokine cocktail. All cytokines were purchased from R&D Systems and PeproTech (Rocky Hill, NJ) and antibodies from eBioscience, BioLegend, and Bio-X-Cell.

### 2.3. RNA Isolation and Quantitative RT-PCR (RT-qPCR)

Cells were collected and total RNA was extracted using TRIzol (Invitrogen). Five hundred ng of RNA was reverse-transcribed into cDNA using the SuperScript III Kit (Invitrogen) according to the manufacturer's protocols. The resulting cDNA template was subjected to quantitative RT-PCR using CFX96 Quantitative RT-qPCR Detection System (Bio-Rad) with SYBR Green Reagent Kit (Invitrogen) according to the manufacturer's protocols. The levels of HuR and other transcripts in activated and Th17 cells were normalized to the levels of GAPDH for each sample. The following primers for specific murine target genes were used in this study: CD3g forward: ACTGTAGCCCAGACAAATAAAGC, CD3g reverse: TGCCCAGATTCCATGTGTTTT; CD3z forward: AAGTGGAAAGTGTCTGTTCTCG, CD3z reverse: TCCATCTAGCAAGTAGCAGAGT; IL-6R*α* forward: TACGCTAGTGACACTTTCTCACA, IL-6R*α* reverse: TTCCGCTTTTTGCCTGAAGTC; IL-17F forward: TGCTGAATGGCGACGGAGTTC, IL-17F reverse: CTGGAGGATAACACTGTGAGAGT; c-Maf forward: AAATACGAGAAGCTGGTGAGCAA, C-Maf reverse: CGGGAGAGGAAGGGTTGTC; and Malt1 forward: GGACAAAGTCGCCCTTTTGAT, Malt1 reverse: TCCACAGCGTTACACATCTCA. Other forward and reverse primers for specific murine were published previously [24–26].

### 2.4. Western Blotting

Whole cell lysates were prepared and western blot analysis was performed as previously described [24, 25]. For phosphorylation protein sample preparation, Th0 and Th17 cells were cultured as described above for 3 days, and then, the cells were rested in T cell culture medium with IL-2 (5 ng/ml) for 2 days; finally, the rested Th0 cells were restimulated with anti-CD3 (5 ng/ml) plus anti-CD28 (2 ng/ml) for 0 to 30 minutes; and the rested Th17 cells were restimulated with TGF-*β* (1 ng/ml) plus IL-6 (20 ng/ml) for 0 to 30 minutes. The concentration of protein was determined with BCA protein assay reagent (Thermo Scientific Pierce, Rockford, IL, USA). Protein samples (whole cell lysates) were sonicated, and 25 *μ*g protein was separated on 10% sodium dodecyl sulphate-polyacrylamide gel electrophoresis (SDS-PAGE) and transferred to a nitrocellulose membrane (Bio-Rad, Hercules, CA, USA). The membranes were blotted with antibodies anti-HuR clone 3A2 (Santa Cruz, CA), anti-p-I*κ*B-*α* (Ser32/36, Santa Cruz Biotech, CA), anti-CD3g (Santa Cruz Biotech, CA), anti-c-Maf (6B8, Santa Cruz Biotech, CA), anti-p-STAT-3, anti-p-Jak1, anti-p-Jak2 (Cell Signaling), and anti-*β*-actin (Santa Cruz Biotech), followed by goat anti-mouse Ig conjugated with HRP secondary antibody (Jackson ImmunoResearch Lab. Inc., West Grove, PA 19390). Membranes were developed by SuperSignal West Pico Chemiluminescent Substrate (Thermo Scientific Pierce, Rockford, IL, USA). Once an image is achieved by scanning the film, it was analyzed by densitometry using Bio-Rad Quantity One software. Briefly, select the region of the band to be quantified and analyze it either automatically or manually. The relative levels of protein expression were obtained by comparing the ratios of detected protein intensities with *β*-actin or *β*-tubulin intensities. The level of protein in baseline was designated as 1 serving as a reference for the particular protein at different conditions.

### 2.5. Intracellular Cytokine Staining

Th17-polarized cells were incubated for 4 hours with 50 ng/ml PMA (Sigma), 500 ng/ml ionomycin (Sigma), and 10 *μ*g/ml brefeldin A (Invitrogen). Cells were stained for surface markers, fixed in 2% formaldehyde, permeabilized with 0.2% saponin, and then stained for intracellular cytokines. Data were collected with BD FACSARIA fusion flow cytometry (BD Biosciences) and analyzed by using FlowJo software (v10, TreeStar).

### 2.6. RNA Immunoprecipitation (RIP)

RIP was performed according to an established protocol [24, 32, 33]. Briefly, Th17-polarized cells were lysed using polysome lysis buffer [33]. Beads (100 *μ*l) were coated by adding 30 *μ*g of either IgG1 (BD Biosciences) as control or anti-HuR antibody (3A2) and incubated at 4°C overnight. After extensively washing the beads, 125 *μ*l of lysate was added and incubated for 4 h at 4°C with additives, and then, 0.5 mg/ml of proteinase K was added and incubated for 30 min at 55°C to digest protein. After extraction, RNA was reverse-transcribed (RT) and amplified by RT-qPCR analysis to assess the presence of specific target mRNAs [24].

### 2.7. Polysome Fraction and Translation Assay

The pellets of WT and HuR KO Th17 cells were lysed using polysome extraction buffer (20 mmol/l Tris-HCl, pH 7.5, 100 mmol/l KCl, 5 mmol/l MgCl_2_, 0.3% Igepal CA-630, protease inhibitors, and 0.1 mg/ml cycloheximide). After centrifugation to remove insoluble material, the lysate was overlaid on a 10% to 50% sucrose gradient. After ultracentrifugation at 39,000 rpm at 4°C, 1 ml fractions were obtained on a density gradient fractionation system. Absorbance (*A*_254_) was measured during the entire fractionation process. Total RNA was extracted from each fraction using TRIzol (Invitrogen, Thermo Fisher Scientific) and analyzed by RT-qPCR analysis.

### 2.8. Statistical Analysis

The Student *t*-test was used to analyze the differences between two groups for the in vitro experiments. The data are expressed as the mean ± SEM. A *p* value < 0.05 was considered statistically significant.

## 3. Results

### 3.1. HuR Strengthens TCR Signaling Pathways of Activated CD4^+^ T Cells

**Figure 1:**
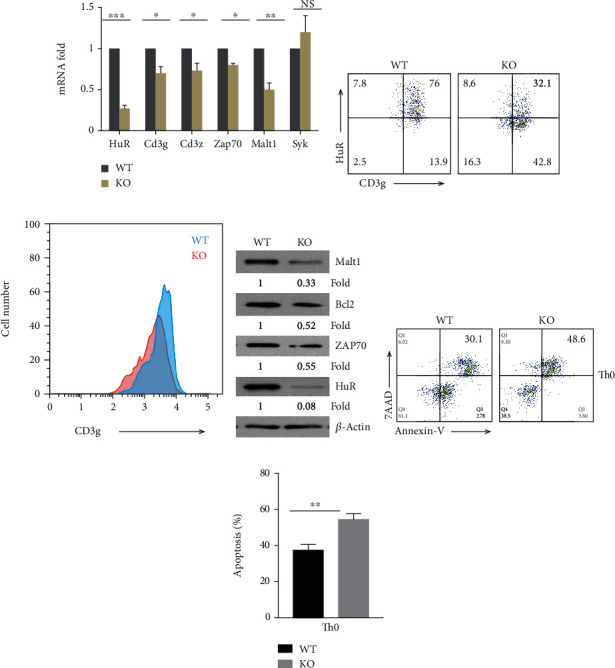
HuR modulates TCR/CD3 complex signaling and protects activated CD4^+^ T cells from apoptosis. Naïve CD4^+^ T cells from the spleen of WT and HuR KO mice were isolated and cultured under Th0 cell polarization conditions as described in Materials and Methods. The polarized cells were collected for further experiments at 5 days after culture. (a) Total RNAs were extracted and transcribed into cDNA. mRNA levels were analyzed by RT-qPCR. (b, c) Expression of HuR and Cd3g was measured by flow cytometric analysis at 4 days of Th0 culture. One of the three repeat experiments is shown. (d) Knockout of HuR decreased abundance of Zap70, Malt1, and Bcl-2, as detected by western blot assay. (e) HuR KO Th0 cells were more susceptible to apoptosis than WT Th0 cells as determined by flow cytometry assay. (f) Summary of three independent experiments is shown for detecting apoptosis by flow cytometry analysis. Data in (a) represents the summary of three independent experiments (mean ± SEM). Data in (b)–(e) represent one of the three independent experiments. Data in (f) represent the summary of the three independent experiments (mean ± SEM) of Th0 cell apoptosis flow cytometric assay. Student's *t*-test was used for statistics analysis. ^∗^*p* < 0.05 and ^∗∗^*p* < 0.01.

Previous study reported that the abundance of HuR was increased following CD4^+^ T cell activation [24, 34]. This suggested that HuR plays a role in T cell activation. Although knockout of HuR reduces the expression of CD3*ζ* in human CD4^+^ T cells [35], the effect of HuR on mouse TCR CD3 complex and its downstream signal pathway has not been investigated. Here, we compared HuR-deficient CD4^+^ T cells from conditional knockout (KO) mice with WT CD4^+^ T cells under anti-CD3 and anti-CD28 stimulation for Th0 cell differentiation, and we identified that the expression of several molecules in the signaling pathway of TCR such as CD3g, Zap70, and Malt1 was impaired in HuR-deficient Th0 cells by RT-qPCR assay (Figure 1(a)). Flow cytometric analysis showed that Cd3g expression was reduced in HuR KO CD4^+^ T cells during Th0 cell differentiation in comparison with WT control cells (Figures 1(b) and 1(c)). Western blot assays showed that the protein levels of Zap70 and Malt1 had decreased (Figure 1(d)), which is consistent with a previous study that HuR KO CD4^+^ T cells are less activated and proliferate less than WT control cells [24]. To determine if HuR protected CD4^+^ T cells from apoptosis, we used flow cytometry to examine the apoptosis of WT and HuR KO Th0 cells. The results showed that the numbers of apoptotic HuR KO Th0 cells significantly increased compared with WT Th0 cells (Figures 1(e) and 1(f)).

**Figure 2:**
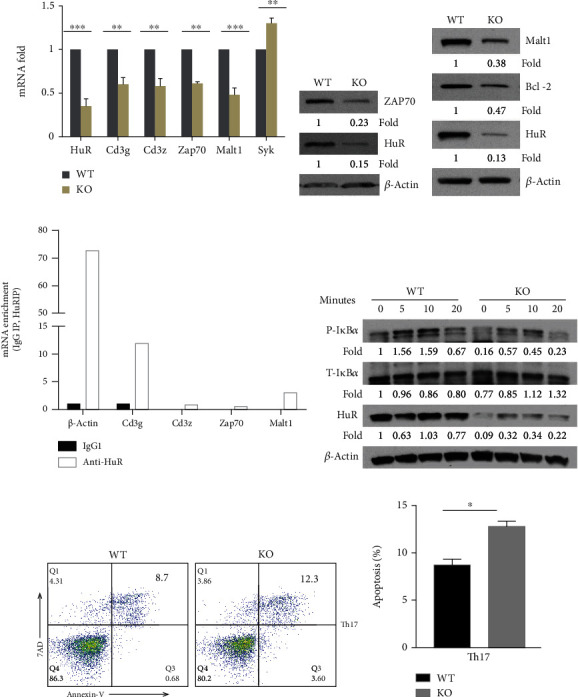
HuR modulates TCR/CD3 complex signaling in Th17 cells. Naïve CD4^+^ T cells from the spleen of WT and HuR KO mice were isolated and cultured under Th17 cell polarization conditions as described in Materials and Methods. The polarized cells were collected for further experiments at 4-5 days after culture. (a) Total RNAs were extracted and reversely transcribed into cDNA. The mRNA levels were analyzed by RT-qPCR. (b, c) Knockout of HuR decreased Zap70, Malt1, and Bcl-2 abundances detected by western blot assay. (d) RIP assay was performed to determine if CD3g, Zap70, and Malt1 mRNAs were associated with HuR protein in Th17 cells. One representative experiment result is known here. The repeated RIP experiment also got the same tendency of results. (e) Naive WT and HuR KO CD4^+^ T cells were cultured at Th17 cell polarization condition for 3 days. Then, Th17 cells were rested 2 days in the presence of IL-2 (3 ng/ml) following restimulation by Th17 cell-polarizing cytokines (TGF-*β*+IL-6) without IL-23 for the indicated times. The level of phosphorylated I*κ*B*α*, but not total I*κ*B*α*, was decreased in HuR KO CD4^+^ T cells compared to WT control cells at indicated time points. The levels of proteins were detected by western blot assay. (f) There is a little difference between HuR KO Th17 cells and WT Th17 cells susceptible for apoptosis as determined by flow cytometry assay. (g) Summary of three independent experiments was shown for apoptosis detected by flow cytometry analysis. Data in (a) represents the summary of three independent experiments (mean ± SEM). Student's *t*-test was used for statistics analysis. ^∗^*p* < 0.05 and ^∗∗^*p* < 0.01. Data in (b)–(e) represents one of the three independent experiments.

We also observed a similar reduced expression of CD3g, Malt1, and Zap70 in HuR KO Th17 cells compared with WT Th17 cells (Figures 2(a)–2(c)). We further performed an RNA immunoprecipitation (RIP) assay to determine how HuR modulated expression of CD3g, Zap70, and Malt1 in Th17 cells. The results showed that CD3g mRNA was highly associated with HuR protein, Malt1 mRNA only modestly, and CD3z and Zap70 mRNA barely associated with HuR protein (Figure 2(d)), suggesting that CD3g is a direct target of HuR. Interestingly, the phosphorylation of I*κ*B*α* was reduced in HuR KO Th17 cells compared with WT control cells (Figure 2(e)), indicating that HuR deficiency blocked activation of NF-*κ*B. However, the numbers of apoptotic HuR KO Th17 cells only slightly increased in comparison with WT Th17 cells (Figures 2(f) and 2(g)). It is well known that Bcl-2 protects various cell types from apoptosis [36]. During T cell development, the expression of Bcl-2 is tightly regulated, which suggests a critical role of Bcl-2 in T lymphocytes [36]. To determine if HuR promoted Bcl-2 expression in CD4^+^ T cells and prevented them from apoptosis, we compared the expression of Bcl-2 in HuR KO and WT Th0 and Th17 cells by western blot assay. The results showed that knockout of HuR significantly reduced Bcl-2 expression in both Th0 and Th17 cells (Figures 1(d) and 2(c)),

Thus, these results suggest that HuR is a positive regulator for CD4^+^ T cell activation. HuR deficiency impairs CD4^+^ T cell activation and differentiation, and HuR-deficient Th0 cells are more susceptible to apoptosis than WT control cells.

### 3.2. Genetic Deletion of HuR Reduces the Activity of Transcription Factor STAT3 in Th17 Cells

**Figure 3:**
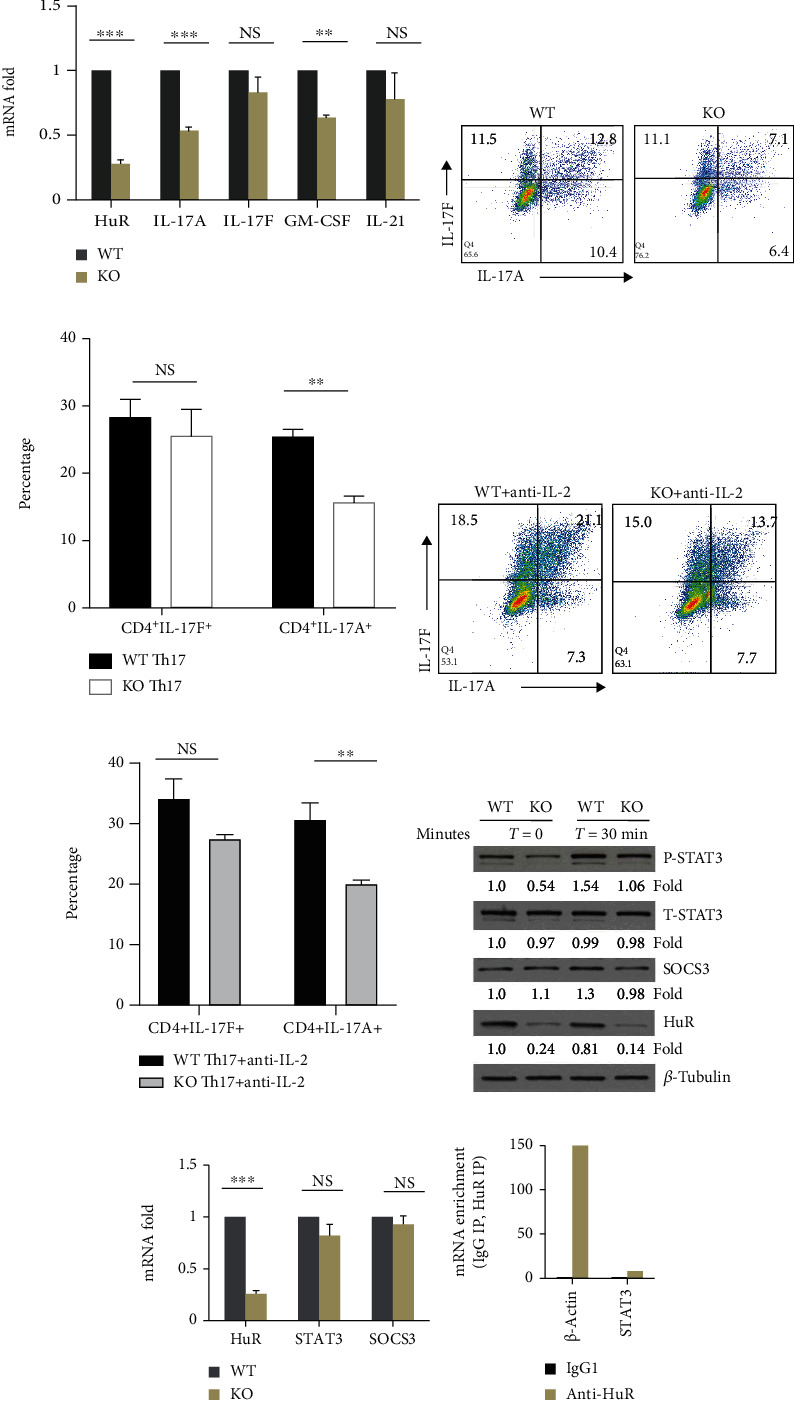
Genetic deletion of HuR reduces the activities of STAT-3 in Th17 cells. (a) Naïve CD4^+^ T cells were stimulated under Th17 cell polarization condition for 4-5 days. The levels of cytokine mRNAs were measured by RT-qPCR. (b) Flow cytometry assay showed that percentage of IL-17A-positive cells significantly decreased, but that of IL-17F-positive cells slightly decreased. (c) Summary of flow data for Th17 cell culture from three independent experiments (mean ± SEM). (d) WT and HuR KO Th17 cells were cultured under Th17 cell culture condition with anti-IL-2 (30 *μ*g/ml) for 5 days. Representative flow cytometric data was shown. (e) Summary of flow data for Th17 cell culture with or without anti-IL-2 from three independent experiments (mean ± SEM). (f) Th17 cells were rested 2 days in the presence of IL-2 (3 ng/ml) following restimulation in the presence of Th17 cell-polarizing cytokines (TGF-*β*+IL-6) without IL-23 for the indicated times. Western blot assay showed that HuR deficiency in Th17 cells reduced the level of p-STAT3 compared to WT Th17 cells, but not total STAT-3. (g) Knockout of HuR did not reduce the level of Stat3 and SOCS3 mRNA in Th17 cells as characterized by RT-qPCR. (h) RIP assay indicated that HuR did not bind to Stat3 mRNA. Data in (a), (c), (e), and (g) represents the summary of three independent experiments. Student's *t*-test was used for statistics analysis. ^∗^*p* < 0.05, ^∗∗^*p* < 0.01, and ^∗∗∗^*p* < 0.001. Data in (b), (d), (f), and (h) represent one of the three independent experiments.

Our previous studies showed that HuR posttranscriptionally regulated IL-17A and GM-CSF mRNA expression by directly binding and stabilizing their mRNAs [24, 26], and the percentage of IL-17A- and GM-CSF-positive Th17 cells is much reduced in HuR KO CD4^+^ T cells compared with WT control cells [26]. Recently, we examined the mRNA expressions of IL-17F and IL-21 by RT-qPCR in both WT and HuR KO Th17 cells. Knockout of HuR merely decreased IL-17F and IL-21 mRNA levels, but significantly decreased IL-17A mRNA level (Figure 3(a)). Flow cytometry analysis showed that IL-17F and IL-17A cytokine production was consistent with their mRNA expressions measured by RT-qPCR analysis (Figures 3(b) and 3(c)) [24]. Previous studies show that HuR restrains IL-2 production in Th2 cells and that IL-2 production is much more increased in HuR KO activated CD4^+^ T cells than WT counterparts [37]. However, the expressions of IL-2 mRNA and protein in HuR KO Th17 cells are comparable with those in WT Th17 cells [24]. It is well known that IL-2 inhibits Th17 cell differentiation [38]. To determine if knockout of HuR-reduced IL-17 production was due to IL-2, we cultured WT and HuR KO CD4^+^ T cells under Th17 cell culture condition with or without anti-IL-2 (30 *μ*g/ml). As expected, neutralization of IL-2 increased IL-17 production in both WT and HuR KO Th17 cells compared to the same Th17 cell culture without IL-2 neutralization. However, WT Th17 cells still produced more IL-17 cytokine than HuR KO Th17 cells in culture when IL-2 was neutralized by anti-IL-2 (Figures 3(d) and 3(e)), suggesting that HuR deficiency impairing IL-17 production was not due to increasing IL-2 production. We speculated that HuR may regulate the activities of transcription factors to promote naïve CD4^+^ T cell differentiation into Th17 cells. To address this question, we performed western blots to detect the activation of transcription factor STAT-3 in HuR KO and WT Th17 cells. The data showed that knockout of HuR did not impair the expression of total STAT-3, but reduced the level of phosphorylation of STAT-3 (Figure 3(f)). SOCS3 is the inhibitor of activation of Stat3 [39, 40]. We hypothesized that knockout of HuR increased expression of SOCS3 to inhibit activation of Stat3. To determine if SOCS3 plays a role in HuR regulating activation of Stat3, we also compared the level of SOCS3 between WT and HuR KO Th17 cells (Figure 3(f)). However, knockout of HuR did not increase but slightly decreased expression of SOCS3 (Figure 3(f)); therefore, it was unlikely that knockout of HuR reduced phosphorylation of Stat3 due to increasing expression of SOCS3. These results were consistent with the RT-qPCR analysis data (Figure 3(g)). To further determine whether HuR bound to STAT3 mRNA, we did RNA immunoprecipitation (RIP) (Figure 3(h)) and STAT3 mRNA decay assay to test its half-life (data not shown) [24]. These data showed that HuR did not directly regulate STAT3 and ROR*γ*t expression [41]. These results suggested that HuR plays a critical role to promote Th17 cell differentiation by upregulating the activity of transcription factor STAT-3.

### 3.3. IL-6R*α* Is a Direct Target of HuR in Th17 Cells

**Figure 4:**
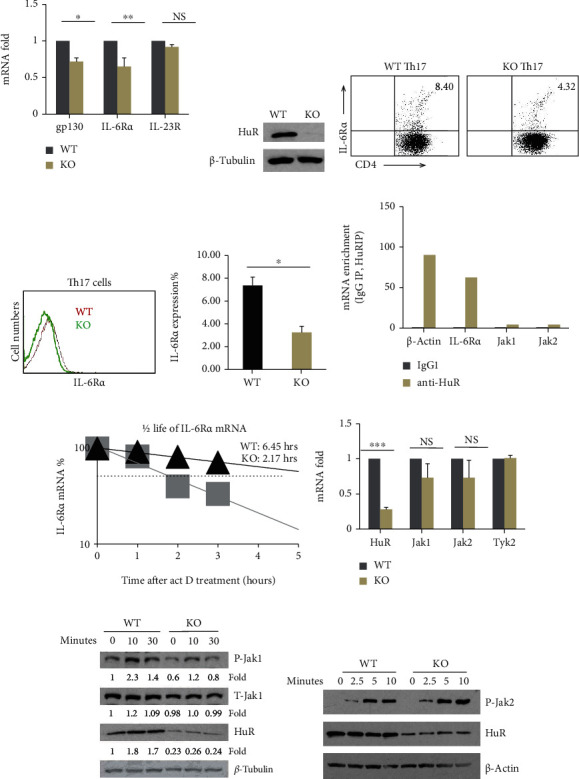
IL-6R*α* is a direct target of HuR. Naïve CD4^+^ T cells were stimulated under Th17 cell-polarizing cytokines for 5 days. (a) The expression of IL-6R*α* and gp130 mRNAs in WT and HuR KO Th17 cells for 4 days of culture was measured by RT-qPCR. (b) The data of western blot assay confirmed that HuR protein was deleted in HuR KO Th17 cells. (c, d) The result of flow cytometry assay showed that IL-6R*α* protein level decreased in KO Th17 cells compared with WT Th17 cells. (e) Summary of IL-6R*α-*positive cells (percentage) from three independent flow cytometric assay experiments is shown (mean ± SEM). (f) RIP assay showed that HuR directly bound to IL-6R*α*, but not Jak1 and Jak2 mRNA. One RIP assay result is shown here. The repeated RIP assay got the same tendency of results but data are not shown here. (g) IL-6R*α* mRNA half-life was much shorter in HuR KO Th17 cells than in WT Th17 cells, as determined by actinomycin D treatment following RT-qPCR assay. One of the two repeat experiments is shown. (h–j) There was no remarkable difference in Jak1 and Jak2 mRNA and protein abundance between WT and HuR KO Th17 cells. (i) The levels of p-Jak1 decreased in HuR KO Th17 cells at the indicated time, (j) but not p-Jak2. Data in (a), (e), and (h) represent the summary of three independent experiments. Student's *t*-test was used for statistics analysis. ^∗^*p* < 0.05, ^∗∗^*p* < 0.01, and ^∗∗∗^*p* < 0.001. Data in (b)–(d) represent one of the three independent experiments. Data in (f), (g), (i), and (j) represent one of the two independent experiments.

It is well known that IL-6 in the presence of TGF-*β* induces Th17 cell differentiation by activation of Stat3 [42]. IL-6 activates Stat3 via IL-6R*α* chain and gp130-mediated signaling and phosphorylated Jak1 [43, 44]. A more recent study shows that IL-6R*α* is indispensable for IL-6-induced action of Stat3 [45]. Based on computation analysis revealing that the 3′UTR of IL-6R*α* mRNA has several potential HuR-binding sites, we hypothesized that HuR may positively regulate IL-6R*α* expression to promote activation of Stat3 during Th17 cell differentiation. To test this hypothesis, we compared the IL-6R*α* mRNA expression between HuR KO and WT Th17 cells by RT-qPCR analysis. The results showed that knockout of HuR significantly reduced the expression of IL-6R*α* mRNA in HuR KO Th17 cells compared with WT control cells (Figures 4(a) and 4(b)). As expected, flow cytometry analysis indicated that the level of IL-6R*α* protein was also reduced in HuR KO Th17 cells (Figures 4(c)–4(e)), which was consistent with the results of RT-qPCR assay.

To understand the mechanism by which HuR modulated IL-6R*α* expression, we further did RIP assay. The data showed that IL-6R*α* mRNA was much enriched in anti-HuR immunoprecipitation (IP) complex but not in the isotype control IgG IP complex, implicating that IL-6R*α* mRNA was physically associated with HuR protein but not Jak1/2 (Figure 4(f)). To determine whether HuR protected IL-6R*α* mRNA from degradation, we treated the cells with actinomycin D as previously described [24, 25]; the level of IL-6R*α* mRNA in HuR KO and WT Th17 cells was measured by RT-qPCR (Figure 4(g)). The results showed that the half-life of IL-6R*α* mRNA in HuR KO Th17 cells was significantly reduced compared with that of WT Th17 cells, suggesting that HuR stabilized IL-6R*α* mRNA. Collectively, these results indicated that HuR directly promotes IL-6R*α* mRNA expression by binding to and stabilizing it and prevents it from decay.

### 3.4. The Downstream Signaling of IL-6/IL-6R Is Impaired in HuR KO Th17 Cells

Cytokine IL-6 binds to IL-6R*α* and gp130 complex and it activates Jak1 and Jak2, which further phosphorylates Stat3 [46], and phosphorylated Stat3 translocate into the nucleus to activate target gene expression [46]. To determine whether HuR deficiency reduced IL-6R*α* expression impairing IL-6/IL-6R downstream signaling pathways, which in turn, reduced activation of Stat3 during Th17 cell differentiation, RT-qPCR was performed to evaluate the immediate IL-6R*α* downstream adaptor Jak1/2 and Tyk2 mRNA levels (Figure 4(g)). Western blot analysis showed that the level of phosphorylation of Jak1 was significantly reduced in HuR KO Th17 cells comparing with WT Th17 cells (Figure 4(i)), but not phosphorylation of Jak2 (Figure 4(j)). Thus, HuR deficiency disrupted the IL-6/IL-6R signaling pathway as characterized by reduced phosphorylation of Jak1 (Figure 4(i)) and Stat3 (Figure 3(f)), leading to at least partly impaired Th17 cell differentiation.

### 3.5. HuR Deficiency Increases IL-22 Production in Th17 Cells by Reducing IL-22 Transcription Suppressor c-Maf Expression

**Figure 5:**
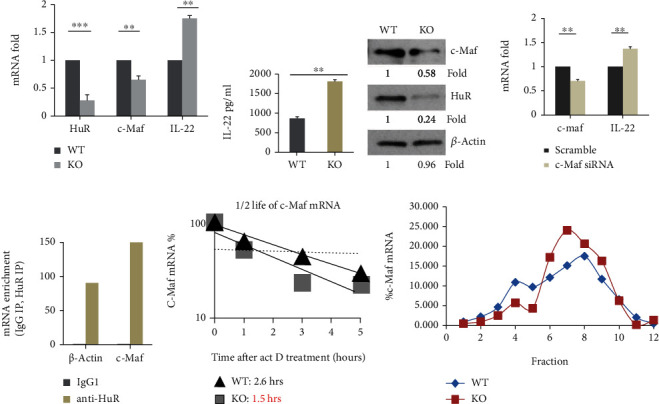
HuR deficiency increases IL-22 production in Th17 cells by reducing c-Maf expression. Naïve CD4^+^ T cells from WT and HuR KO mice were cultured under Th17 cell polarization condition for 5 days. (a) The expression of c-Maf and IL-22 mRNAs was examined by RT-qPCR. IL-22 mRNA RT-qPCR is a positive control in (a). (b) The levels of IL-22 in WT and HuR KO Th17 cell culture supernatant were examined by ELISA. (c) The abundance of c-Maf and HuR protein in WT and HuR KO Th17 cells was examined by western blots. (d) Naive WT CD4^+^ T cells were transduced with c-Maf siRNA and scramble siRNA and polarized under Th17 cell culture condition for 5 days. The expression of IL-22 and c-Maf mRNA was measured by RT-qPCR assay. (e) HuR bound to c-Maf mRNA as determined by RIP assay; one of the two repeated experiments is shown. The repeated RIP experiment got the same tendency of results. (f) Actinomycin D (3 *μ*g/ml)-treated Th17 cells were harvested at 1, 3, and 5 h; the level of c-Maf mRNA was determined by RT-qPCR assay. One of the two repeated experiments is shown. (g) Polysome fraction assay showed that HuR did not modulate c-Maf mRNA translation. Results in (a) and (b) represent the summary of three independent experiments (mean ± SEM). Student's *t*-test was used for statistics analysis. ^∗∗^*p* < 0.01 and ^∗∗∗^*p* < 0.001. Data in (c)–(g) represent one of the two independent experiments.

It is well known that Th17 cells not only produce cytokines IL-17A, IL-17F, GM-CSF, and IL-21 but also produce IL-22 [1]. Knockout of HuR significantly reduces the expression of IL-17A and GM-CSF [25, 26] and slightly reduces IL-17F and IL-21 (Figure 3(a)). However, we found that the abundance of IL-22 mRNA was increased in HuR KO Th17 cells compared with WT Th17 cells *in vitro* (Figure 5(a)) [24], which was consistent with increased IL-22 cytokine in culture supernatants of HuR KO Th17 cells compared with WT cells, as determined by ELISA (Figure 5(b)). Given that TGF-*β* inhibits IL-22 production by promoting suppressor c-Maf expression [30], we hypothesized that HuR deficiency reduced the abundance of c-Maf resulting in the upregulated IL-22 expression. To test this hypothesis, we compared the abundance of c-Maf in HuR KO Th17 cells with that of WT Th17 cells. As expected, the expressions of c-Maf mRNA and protein decreased in HuR KO Th17 cells compared with WT Th17 cells (Figures 5(a) and 5(c)). To confirm if c-Maf is a negative transcription factor for IL-22 in our culture system, we electrically transfected WT Th17 cells with c-Maf siRNA and scrambled siRNA as a control. The data showed that knockdown of c-Maf increased IL-22 mRNA expression (Figure 5(d)). Thus, the expression of c-Maf inversely correlated with the expression of IL-22 in HuR-deficient and WT Th17 cells.

To explore how HuR regulated c-Maf expression, we did RIP and mRNA decay assay. The results showed that c-Maf mRNA was highly enriched with anti-HuR IP complex (Figure 5(e)), suggesting that HuR bound to c-Maf mRNA. The half-life of c-Maf mRNA was significantly shorter in HuR KO Th17 cells than WT Th17 cells (Figure 5(f)), suggesting that HuR protected c-Maf mRNA from degradation. Translation assay showed that HuR had no significant impact on c-Maf mRNA translation (Figure 5(g)). These results suggested that HuR binds to and stabilizes c-Maf mRNA leading to increased abundance of c-Maf protein, which, in turn, limits production of IL-22. Therefore, HuR deficiency decreased c-Maf expression but increased IL-22 production.

## 4. Discussion

Previous studies focused on the function of HuR in controlling cancer cell growth, proliferation, and metastasis [20, 47]; however, few studies were about the role of HuR in T lymphocyte activation [21, 48, 49]. Although cytokine-mediated transcriptional regulation of CD4^+^ T cell activation and differentiation has been extensively investigated [11, 19, 50], posttranscriptional regulation by HuR in CD4^+^ T cell activation remains unclear. Here, we provided evidences that HuR promoted Zap70 and CD3g expression. HuR directly bound to and protected CD3g mRNA from decay. However, HuR did not bind to mouse CD3z mRNA, which is different from the function of HuR binding to human CD3z mRNA as previously reported [35]. Although knockout of HuR decreased Zap70 mRNA and protein levels, HuR did not directly modulate the expression of ZAP70. Our study also showed that HuR impaired the downstream Malt1 and phosphorylated I*κ*B*α* expression. How HuR regulate ZAP70 and Malt1 expression will be addressed in future study. Based on Malt1 and activation of NF-*κ*B contributing to Th17 cell differentiation [9, 51], we speculated that the impaired TCR signaling due to HuR deficiency partially led to impairing Th17 cell differentiation. However, the effect of HuR on the IL-6-IL-6R signaling axis also contributed to Th17 cell differentiation (see below). Interestingly, compared with WT Th0 cells, HuR KO Th0 cells were more susceptible to activation-induced apoptosis; there was little difference between HuR KO and WT Th17 cells susceptible to apoptosis (Figures 2(f) and 2(g)). These results are in line with previous works that Th17 cells are less susceptible to apoptosis than other T helper subsets [52, 53]. Although western blot assay showed that the expression of Bcl-2 was reduced in both HuR KO Th0 and Th17 cells, knockout of HuR did not significantly increase Th17 cell susceptibility to apoptosis than WT Th17 cells, which may be due to highly expressed c-Flip in Th17 cells for their resistance to apoptosis [52, 53].

IL-2 suppresses Th17 cell differentiation [38]. IL-2 production is much more increased in activated HuR KO CD4^+^ T cells than WT counterparts [49]. However, WT Th17 cells still produced more IL-17 cytokine than HuR KO Th17 cells in culture with IL-2 neutralization, suggesting that HuR deficiency reduced IL-17 production not mainly due to IL-2 for inhibiting Th17 cell differentiation, which is consistent with no significant difference in IL-2 mRNA expression between WT and HuR KO Th17 cells [24].

In the current study, we also found that HuR restrained IL-22 production by promoting Th17 cell transcription factor c-Maf expression, which suppressed IL-22 production. Mechanistically, c-Maf was a direct target of HuR, and HuR bound to and stabilized c-Maf mRNA. We further showed that knockdown of c-Maf by c-Maf siRNA transfection increased expression of IL-22 mRNA in Th17 cells, which is in line with the notion that HuR restrains IL-22 expression by increasing expression of c-Maf.

IL-6/IL-6R is important for Th17 cell differentiation [50, 54], and Stat3 activation is responsible for IL-6-dependent T cell proliferation [55]. IL-6R*α* is indispensable for IL-6-induced phosphorylation of Stat3. Previous work demonstrated that HuR protects IL-6 mRNA from degradation by preventing RNA-binding protein TTP-mediated decay in macrophages [56]. Thus, HuR may positively promote Th17 cell differentiation via multiple mechanisms such as upregulating IL-6R*α* expression on T cells and increasing IL-6 production by antigen-presenting cells [56]. IL-6 is a proinflammatory cytokine that plays a crucial role in several autoimmune diseases including EAE and rheumatoid arthritis [57, 58]. IL-6-deficient mice are resistant to EAE [58], and neutralization of IL-6 reduces the severity of EAE induced by Th17 and Th1 cells [59]. Overall, these results emphasize the significance of HuR in regulating the IL-6/IL-6R signaling axis in T cell activation and disease, which is in line with our recent work that HuR plays an important role in autoimmune neuroinflammation [41].

We have revealed that HuR upregulated IL-6R*α* expression in Th17 cells, which in turn upregulated activity of Jak1, transcription factor Stat3, and ROR*γ*t expressions to enhance Th17 cell differentiation. Although SOCS3 can suppress activation of Stat3 [39], knockout of HuR slightly reduced expression of SOCS3, which is unlikely that HuR promoted phosphorylation of Stat3 via SOCS3. This finding in HuR functions appears unique, because HuR modulated transcription factors for cell differentiation, which is different from the function of HuR on preventing cytokine and chemokine mRNAs from decay [24, 25, 28]. Although recent literature reported that HuR promotes Stat3 translation in muscle cells during inflammation-induced muscle wasting [29], knockout of HuR did not impair total Stat3 expression in mouse Th17 cells. This needs to be further confirmed when new HuR deleting system is available. Our recent work also showed that knockout of HuR reduced the expression of ROR*γ*t in Th17 cells, and HuR did not bind to and stabilize ROR*γ*t mRNA, suggesting that HuR indirectly regulates expression of ROR*γ*t [41]. Given that activation of Stat3 promotes ROR*γ*t expression in Th17 cells [42], we speculated that HuR upregulated activation of Jak1 and Stat3 to promote ROR*γ*t expression in Th17 cells. In addition, our recent work showed that HuR modulates Runx1 expression to promote myelin oligodendrocyte glycoprotein- (MOG-) activated Th17 cell differentiation [41]. Thus, HuR may promote Th17 cell differentiation by activation of Jak1 and Stat3 and expression of ROR*γ*t, Runx1, and c-Maf.

Collectively, HuR modulates CD4^+^ T cell activation and differentiation via distinct mechanisms. The current study provides further understanding of complexity in cellular and molecular regulation of CD4^+^ T cell activation and differentiation by RNA-binding protein HuR.

## Data Availability

All the data are included in this manuscript.
